# Supplementary material for: From sequence to enzyme mechanism using multi-label machine learning
Source: BMC Bioinformatics. 2014 May 19;15:150. doi: 10.1186/1471-2105-15-150 (PMC4229970; doi:10.1186/1471-2105-15-150)
Supplement: Additional file 2 — Java code of ml2db. Additional file ml2db_code.tar.gz contains the Java source code to run the multi-label machine learning experiments and save the results to database. The code’s Javadoc is included. [file 1471-2105-15-150-S2.zip › additional file 2/ml2db/utils/doc/uk/ac/ed/inf/utils/maputils/class-use/OneToManyMap.html]

Uses of Class uk.ac.ed.inf.utils.maputils.OneToManyMap


---


|  |  |  |
| --- | --- | --- |
| **Overview** \| **Package** \| **Class** \| **Use** \| **Tree** \| **Deprecated** \| **Index** \| **Help** | |  |
| PREV   NEXT | **FRAMES**    **NO FRAMES**     **All Classes** |  |


---


## **Uses of Class uk.ac.ed.inf.utils.maputils.OneToManyMap**

| Packages that use OneToManyMap | |
| --- | --- |
| **test.maputils** |  |
| **uk.ac.ed.inf.utils.collectionutils.setutils** |  |
| **uk.ac.ed.inf.utils.database** |  |
| **uk.ac.ed.inf.utils.maputils** |  |
| **uk.ac.ed.inf.utils.setutils** |  |

Uses of OneToManyMap in test.maputils

| Methods in test.maputils that return OneToManyMap | |
| --- | --- |
| `static OneToManyMap<java.lang.String,java.lang.String>` | `OneToManyMapTest.oneToManyMap1()` |
| `static OneToManyMap<java.lang.String,java.lang.String>` | `OneToManyMapTest.oneToManyMap2()` |
| `static OneToManyMap<java.lang.String,java.lang.String>` | `OneToManyMapTest.oneToManyMapEmptyKeyValue()` |

Uses of OneToManyMap in uk.ac.ed.inf.utils.collectionutils.setutils

| Methods in uk.ac.ed.inf.utils.collectionutils.setutils that return OneToManyMap | |
| --- | --- |
| `OneToManyMap` | `SupersetsManager.getSupersetsIds()` |

Uses of OneToManyMap in uk.ac.ed.inf.utils.database

| Methods in uk.ac.ed.inf.utils.database that return OneToManyMap | |
| --- | --- |
| `OneToManyMap<T,U>` | `DbReader.get2ColumnsValues(java.sql.ResultSet resultSet, int columnIndexOfKeys, int columnIndexOfValues, OneToManyMap<T,U> map, int expectedNumberOfKeysUniqueStrings, int expectedNumberOfValuesUniqueStrings)`             Get the nth and mth column of a `ResultSet` |
| `OneToManyMap<T,U>` | `DbReader.getMap(java.lang.String sql, int columnIndexOfKeys, int columnIndexOfValues, int expectedNumberOfKeysUniqueStrings, int expectedNumberOfValuesUniqueStrings)`             Converts a query resultset into a map. |
| `OneToManyMap<T,U>` | `DbReader.queryResultsColumnsToMap(java.lang.String sql, int columnIndexOfKeys, int columnIndexOfValues, int expectedNumberOfKeysUniqueStrings, int expectedNumberOfValuesUniqueStrings)`             From two resultset columns to a key-value map |

| Methods in uk.ac.ed.inf.utils.database with parameters of type OneToManyMap | |
| --- | --- |
| `OneToManyMap<T,U>` | `DbReader.get2ColumnsValues(java.sql.ResultSet resultSet, int columnIndexOfKeys, int columnIndexOfValues, OneToManyMap<T,U> map, int expectedNumberOfKeysUniqueStrings, int expectedNumberOfValuesUniqueStrings)`             Get the nth and mth column of a `ResultSet` |

Uses of OneToManyMap in uk.ac.ed.inf.utils.maputils

| Subclasses of OneToManyMap in uk.ac.ed.inf.utils.maputils | |
| --- | --- |
| `class` | `IndexedOneToManyMap<T,U>`             A one-to-many map that uses an internal integer index to save memory. |

| Methods in uk.ac.ed.inf.utils.maputils that return OneToManyMap | |
| --- | --- |
| `static <T> OneToManyMap<T,T>` | `MapUtils.fromArrayOfArrayToOneToManyMap(T[][] array)`             Array format must be [[key1, value1][key1, value2],[key2, value3],[...]]. |
| `OneToManyMap<java.lang.String,java.lang.String>` | `TableMap.getMap()`             Get the keys to values map |
| `OneToManyMap<java.lang.String,java.lang.String>` | `TableMap.getReverseMap()`             Get the values to keys map |
| `static OneToManyMap<java.lang.String,java.lang.String>` | `OneToManyMap.invertKeysAndValues(OneToManyMap<java.lang.String,java.lang.String> map)` |

| Methods in uk.ac.ed.inf.utils.maputils with parameters of type OneToManyMap | |
| --- | --- |
| `void` | `OneToManyMap.addAll(OneToManyMap<T,U> mapToAdd)`             Add all elements of a one2many map to another map |
| `static OneToManyMap<java.lang.String,java.lang.String>` | `OneToManyMap.invertKeysAndValues(OneToManyMap<java.lang.String,java.lang.String> map)` |

Uses of OneToManyMap in uk.ac.ed.inf.utils.setutils

| Methods in uk.ac.ed.inf.utils.setutils that return OneToManyMap | |
| --- | --- |
| `OneToManyMap` | `SupersetsManager.getSupersetsIds()` |

---


|  |  |  |
| --- | --- | --- |
| **Overview** \| **Package** \| **Class** \| **Use** \| **Tree** \| **Deprecated** \| **Index** \| **Help** | |  |
| PREV   NEXT | **FRAMES**    **NO FRAMES**     **All Classes** |  |


---
